# Simultaneous Quantification of Four Phenylethanoid Glycosides in Rat Plasma by UPLC-MS/MS and Its Application to a Pharmacokinetic Study of Acanthus Ilicifolius Herb

**DOI:** 10.3390/molecules24173117

**Published:** 2019-08-28

**Authors:** Mengqi Zhang, Xia Ren, Shijun Yue, Qing Zhao, Changlun Shao, Changyun Wang

**Affiliations:** 1 Key Laboratory of Marine Drugs, The Ministry of Education of China, School of Medicine and Pharmacy, Ocean University of China, Qingdao 266003, China; 2 Laboratory for Marine Drugs and Bioproducts, Qingdao National Laboratory for Marine Science and Technology, Qingdao 266237, China

**Keywords:** acanthus ilicifolius herb, phenylethanoid glycosides, pharmacokinetics, UPLC-MS/MS

## Abstract

Acanthus ilicifolius herb (AIH), the dry plant of *Acanthus ilicifolius* L., has long been used as a folk medicine for treating acute and chronic hepatitis. Phenylethanoid glycosides (PhGs) are one family of the main components in AIH with hepatoprotective, antioxidant, and anti-inflammatory activities. In this study, the pharmacokinetics of AIH was investigated preliminarily by ultra-performance liquid chromatography coupled with triple quadrupole mass spectrometry (UPLC-MS/MS). A simultaneously quantitative determination method for four PhGs (acteoside, isoacteoside, martynoside, and crenatoside) in rat plasma was first established by UPLC-MS/MS. These four PhGs were separated with an ACQUITY UPLC BEH C_18_ column (2.1 × 50 mm, 1.7 μm) by gradient elution (mobile phase: MeCN and 0.1% formic acid in water, 0.4 mL/min). The mass spectrometry detection was performed using negative electrospray ionization (ESI^−^) in multiple reaction monitoring (MRM) mode. By the established method, the preliminary pharmacokinetics of AIH was elucidated using the kinetic parameters of the four PhGs in rat plasma after intragastric administration of AIH ethanol extract. All four PhGs showed double peaks on concentration-time curves, approximately at 0.5 h and 6 h, respectively. Their elimination half-lives (t_1/2_) were different, ranging from 3.42 h to 8.99 h, although they shared similar molecular structures. This work may provide a basis for the elucidation of the pharmacokinetic characteristics of bioactive components from AIH.

## 1. Introduction

*Acanthus ilicifolius* L. is a mangrove shrub belonging to the *Acanthus* genus in the Acanthaceae family which grows in tropical and subtropical intertidal habitats. Acanthus ilicifolius herb (AIH, “laoshule” in Chinese), the dry plant of *A. ilicifolius* L., is a folk medicine to treat acute and chronic hepatitis, lymphatic intumescence, spleen enlargement, paralysis, and rheumatism [1,2,3,4,5,6]. Modern pharmaceutical studies have demonstrated that the extracts of AIH possess hepatoprotective, antioxidant, anti-inflammatory, anticarcinogenic, and antibacterial activities [6,7,8,9,10]. Numerous chemical constituents have been isolated from AIH, including phenylethanoid glycosides (PhGs), alkaloids, flavones, lignans, triterpenoid saponins, and sterols [11,12,13,14,15,16,17]. Among them, PhGs were reported as one family of the main components from AIH, such as acteoside, isoacteoside, martynoside, and crenatoside with potential pharmacological effects [18,19,20,21,22,23]. The pharmacologic actions of AIH extracts, as well as these four PhGs, have been reported, whereas no pharmacokinetics have been studied till now.

During our ongoing research of pharmacodynamic material basis of medical plants, we found that AIH and its efficient components, PhGs, exhibited potent hepatoprotective, antiviral, and antioxidant activities. Specifically, PhGs were found to be abundant in AIH, of which acteoside and isoacteoside were the main components, while martynoside and crenatoside were in relatively lower contents. In the present study, the pharmacokinetics of AIH were investigated with these four PhGs as representatives in rat plasma after intragastric administration of AIH extract. To achieve this purpose, a simple, sensitive and rapid ultra-performance liquid chromatography coupled with triple quadrupole mass spectrometry (UPLC-MS/MS) method was established, first, for the simultaneous and quantitative determination of the four PhGs in rat plasma. Herein, we report the establishment and optimization of UPLC-MS/MS method, the validation of the established method, and the pharmacokinetic study of AIH. 

## 2. Results and Discussion

### 2.1. Establishment and Optimization of the UPLC-MS/MS Method

**Figure 1:**
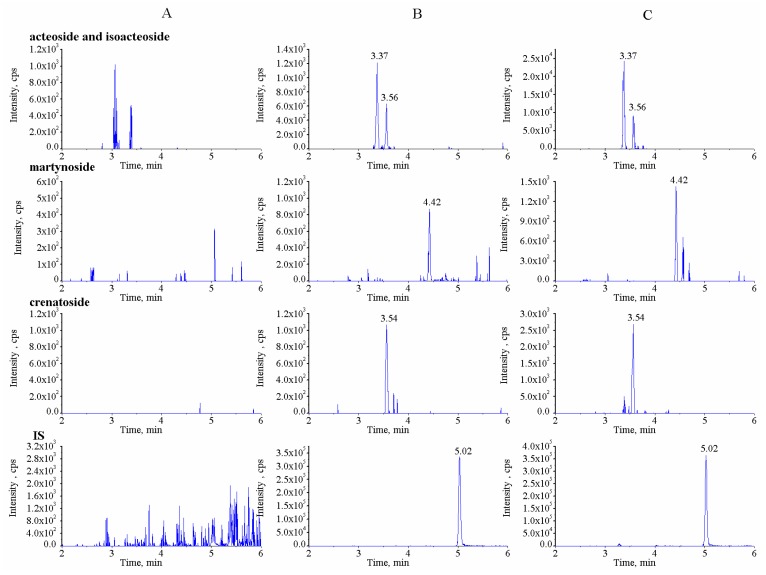
The multiple reaction monitoring (MRM) chromatograms for the four phenylethanoid glycosides (PhGs) and genistein (IS) in (**A**) blank plasma, (**B**) blank plasma spiked with the four PhGs at the lowest limit of quantification (LLOQ) and IS (1000 ng/mL), and (**C**) test plasma collected at 30 min after intragastric administration.

The UPLC-MS/MS method was established and optimized to determine the four PhGs (acteoside, isoacteoside, martynoside, and crenatoside) simultaneously and quantitatively. In preliminary experiments, the two isomer analytes, acteoside and isoacteoside, were found to have the same MS fragmentation characteristics. Therefore, it was required to separate these two analytes from each other by UPLC because of their influence on each other. In our study, different mobile phase compositions were screened, including methanol and water, methanol and 0.1% formic acid in water, MeCN and water, MeCN and 0.1% formic acid in water, and MeCN and 0.5% formic acid in water. It was found that the mobile phase consisting of MeCN and 0.1% formic acid in water significantly improved the peak shapes and achieved the baseline separation of acteoside and isoacteoside (Figure 1). Moreover, in comparison with isocratic elution, the gradient elution with MeCN and 0.1% formic acid shortened analysis duration and increased separation efficiency. 

**Figure 2:**
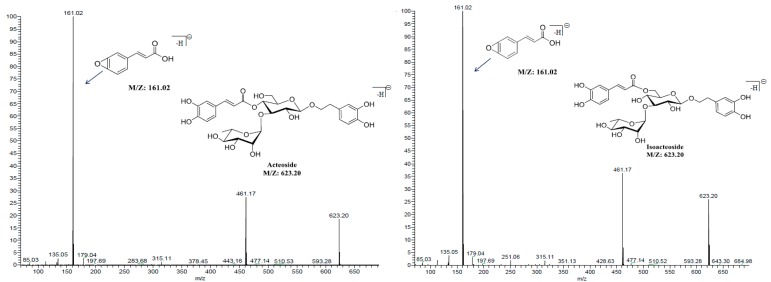

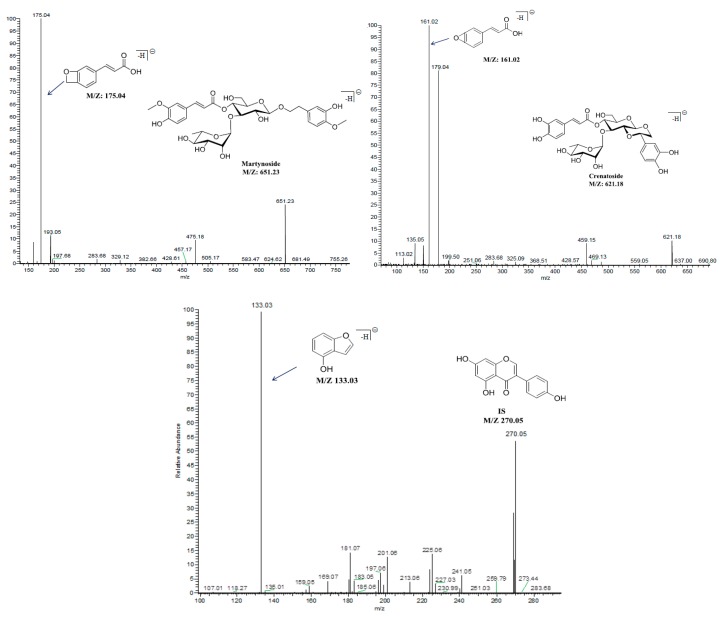
The structures, conjectural fragments, and MS/MS spectra of PhGs and IS at 30 V collision energy.

The mass conditions were modified to find the optimum precursor-to-product ion pairs for MRM detection by the production scan procedure. Both positive and negative ionization ESI modes were tested for the four PhGs and genistein (IS). It was observed that the negative mode (ESI^−^) of the four PhGs achieved better sensitivity as compared with the positive mode. Among them, acteoside, isoacteoside, and crenatoside displayed the same characteristic MS fragments of *m*/*z* 161.02 in the mass spectra due to their similar molecular structures (Figure 2 and Table 1). 

It should be pointed that genistein was selected as the IS because it displayed a strong MS response under negative ion mode (ESI^−^) and presented satisfactory chromatographic behavior (Figure 2 and Table 1).

### 2.2. Contents of the Four PhGs in AIH

The contents of the four PhGs were analyzed by UPLC-MS/MS utilizing the same chromatography method as described in Section 3.6. The contents of acteoside, isoacteoside, martynoside, and crenatoside in AIH ranged from 0.02 to 6.24 mg/g (Table 2).

### 2.3. Method Validation

The established UPLC-MS/MS method was validated for its selectivity, linearity, accuracy, precision, extraction recovery, matrix effect, and stability according to the FDA Guidance for Industry on Bioanalytical Method Validation [24].

#### 2.3.1. Selectivity

Representative chromatograms of the blank plasma sample, blank plasma sample added with PhGs at the lowest limit of quantification (LLOQ) and IS, and treated plasma samples are shown in Figure 1. The results suggest that no significant endogenous interference was found around the retention time of the PhGs and IS. 

#### 2.3.2. Linearity and LLOQ

The concentrations of PhGs in test samples were calculated by the calibration curves, which showed good linearities with *r*^2^ > 0.993. The LLOQ of the four PhGs were determined ranging from 0.2 to 2.0 ng/mL in accordance with the signal-to-noise ratios (S:N) > 10 (Table 3). These results indicated that the linearity and LLOQ were feasible for quantificational detection of the four PhGs in rat plasma. 

#### 2.3.3. Accuracy and Precision

Our results showed that the intra- and inter-day accuracies of the four PhGs ranged from −8.92% to 9.88%, while the precisions ranged from 1.47% to 13.08% (Table 4), indicating that the method had satisfactory accuracy and precision.

#### 2.3.4. Extraction Recovery and Matrix Effect

It was found that the extraction recoveries of the four PhGs ranged from 70.56% to 104.54% at three concentration levels (Table 5). It should be noted that there were large variations of extraction recovery for acteoside and isoacteoside. The reason may be that the extraction recoveries of acteoside and isoacteoside suffered from low to moderate suppression due to matrix effects, which would not limit the use of this method, given the satisfactory precision and reproducibility obtained. 

The absolute matrix effects of these PhGs were from 85.03% to 106.66% (Table 5). And the relative standard deviation (RSD) values of relative matrix effects were less than 10.90%. The IS normalized matrix factors were 0.90–1.04. The above results indicated that the extraction recoveries of these PhGs were reliable, and there was almost no significant matrix effect in this experiment.

#### 2.3.5. Stability

The stability results of the four PhGs at three concentrations under four conditions are summarized in Table 6. These four PhGs were observed to be stable under a variety of storage and process conditions with the RSD values less than 12.48% and the RE values from −11.62% to 4.70%.

### 2.4. Pharmacokinetic Study

The established and validated UPLC-MS/MS method was used to investigate the pharmacokinetics of AIH represented by the four PhGs in rat plasma after intragastric administration of AIH ethanol extract. 

**Figure 3:**
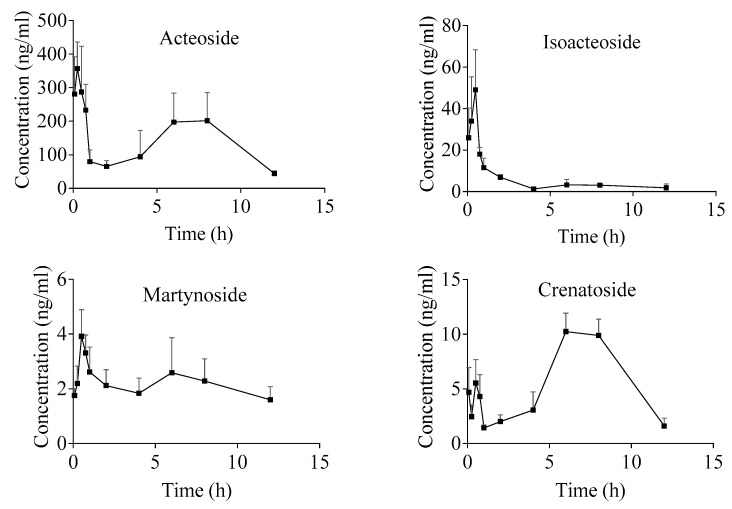
Mean plasma concentration-time curves of the four PhGs in rat plasma (*n* = 6).

We found that all of the four PhGs could be detected from plasma at 5 min after intragastric administration of AIH ethanol extract (Figure 3). All of the PhGs showed double peaks on concentration-time curves. The first concentration peaks of all the PhGs appeared at about 0.5 h, and then, reached the second peaks at approximately 6 h in rat plasma. Interestingly, a literature survey indicated that the concentration-time curve features of acteoside and isoacteoside have been reported as double peaks within 1 h in rat plasma after intragastric administration of individual components [25,26], whereas, they have been reported as single peaks [27,28] or double peaks [26,29] within 2 h when administrated medicinal plant extracts. In our study, the relatively distant double peaks of the PhGs might attribute to multiple reasons, such as the influences of complex compositions in AIH, enterohepatic circulation, multiple absorption sites, and gastric emptying process. 

Among the four PhGs, the concentration of the second peak of crenatoside was higher than that of the first peak, but the concentration of the second peaks of the other three analytes were lower than those of their first peaks. The peak times (t_max_) of acteoside and isoacteoside were at 0.3 ± 0.1 h and 0.4 ± 0.2 h (Table 7), respectively, which were consistent with previous studies [26,28,29,30]. The t_max_ of martynoside and crenatoside were at 3.1 ± 3.6 h and 6.8 ± 1.1 h, respectively, which were longer than those of acteoside and isoacteoside.

The areas under the curves (AUC_0–t_) of the four PhGs were consistent with their contents in AIH, for example, acteoside exhibited the highest AUC_0–t_ as 1826.3 ± 680.2 µg/L × h and the highest content up to 6.24 mg/g. The four PhGs displayed different elimination half-lives (t_1/2_), ranging from 3.4 h to 9.0 h, although they have similar molecular structures. 

## 3. Materials and Methods 

### 3.1. Chemicals and Reagents

The authentic phenylethanoid glycoside compounds were purchased as follows: acteoside (purity 98.0%) from Dalian Meilun Biological Technology Co., Ltd. (Dalian, China); isoacteoside (purity 98.0%) from Chengdu Push Bio-Technology Co., Ltd. (Chengdu, China); and genistein (internal standard, IS) from Shanghai Aladdin Biochemical Technology Co., Ltd. (Shanghai, China). Martynoside and crenatoside (>98.0% purity) were isolated from AIH in our laboratory and identified by combination of NMR, HPLC, and MS. HPLC-grade acetonitrile (MeCN) and methanol were purchased from Fisher Scientific Co., Ltd. (St. Louis, MO, USA). HPLC-grade formic acid was obtained from Shanghai Macklin Biochemical Co., Ltd. (Shanghai, China), and experimental water was purified by a Milli-Q Reagent Water System (Millipore, Burlington, MA, USA).

### 3.2. Preparation of AIH Extracts 

The plant *A. ilicifolius* L. was collected from Jiangmen, Guangdong Province, China and authenticated by Professor Feng-Qin Zhou, Shandong University of Traditional Chinese Medicine. Voucher specimen number for *A. ilicifolius* L. is 2018060805. Voucher specimen of the plant is deposited at the Key Laboratory of Marine Drugs, the Ministry of Education of China, Ocean University of China, Qingdao, China.

The whole plant was dried in the shade and ground into crude powder. The crude powder (200 g) was immersed in 95% ethanol (*v*/*w*, 10:1) for 1 h, and then heated to reflux at 80 °C for 2 h. The extraction solution was filtered, and the residue was refluxed again in 95% ethanol (*v*/*w*, 8:1) at 80 °C for 2 h. The filtrate was pooled together and concentrated by a rotary evaporator to dryness at 45 °C. Finally, the product was dissolved in distilled water to acquire the AIH extract for testing with the concentration of 5.0 g crude herb/mL. This extract sample was stored at 4 °C until use.

### 3.3. Animals 

Ten male Sprague-Dawley (250–280 g) rats were purchased from Jinan Pengyue Experimental Animal Center (SCXK (Lu) 20140007). The animal experiments were approved by the Animal Ethics Committee of Marine Biomedical Research Institute of Qingdao (MBRI-2018-0606), and the guidelines of the institute were strictly followed. All rats had free access to water and food, and were maintained in an environmentally controlled breeding room under the following conditions: 20 ± 2 °C temperature, 60–70% relative humidity, and 12 h light/dark for 1 week before the experiment operated. After fasted for 12 h with free access to water, blank plasma was obtained from four rats after intragastric administration of 2.0 mL/kg water for the UPLC-MS/MS method validation, and the other six rats which were administrated with AIH extract were used for the pharmacokinetic study as in Section 2.4. 

### 3.4. Preparation of Stock Solutions, Calibration Samples, and Quality Control Samples

The stock solutions of four PhGs, acteoside, isoacteoside, martynoside, and crenatoside, were prepared with methanol as a solvent. A stock solution of the PhGs mixture was prepared by combining these four PhGs to attain the final concentrations of 10,000 ng/mL acteoside, 1000 ng/mL isoacteoside, 2000 ng/mL martynoside, and 2000 ng/mL crenatoside. The working solutions were obtained from the stock solution by sequential dilution with methanol at the concentrations of 20.0–10,000 ng/mL acteoside, 2.0–1000 ng/mL isoacteoside, 4.0–2000 ng/mL martynoside, and 4.0–2000 ng/mL crenatoside. Calibration samples were prepared by adding 5 μL working solutions to 45 μL blank plasmas in 1.5 mL Eppendorf tubes. Therefore, the final calibration samples contained 2.0–1000 ng/mL acteoside, 0.2–100 ng/mL isoacteoside, 0.4–200 ng/mL martynoside, and 0.4–200 ng/mL crenatoside. The quality control (QC) samples were prepared in the same way as calibration samples, with the final dilutions of 5.0, 50, and 800 ng/mL acteoside, 0.5, 5, and 80 ng/mL isoacteoside, 1.0, 10, and 160 ng/mL martynoside, and 1.0, 10, and 160 ng/mL crenatoside. The stock solution of the internal standard (IS), geistein, was prepared in methanol at the concentration of 1.0 mg/mL. The IS working solution of 1000 ng/mL was obtained by diluting the stock solution with methanol.

### 3.5. Pretreatment of Calibration Samples and QC Samples 

The 10 μL IS working solution and 140 μL methanol were added to each calibration sample (50 μL) and QC sample (50 μL). The mixture was vortexed for 60 s and centrifuged at 15,000× *g* for 15 min to separate the precipitated protein. Then, 2 μL of supernatant of the mixture was used for the UPLC-MS/MS analysis.

### 3.6. Instrumentation and Chromatographic Conditions

A Waters ACQUITY™ UPLC system (Waters Corp., Milford, MA, USA) was interfaced with a Waters Xevo™ TQ/MS (Waters, USA) equipped with an electrospray ionization (ESI) source. Separation of the PhGs was performed on an ACQUITY UPLC BEH C_18_ column (2.1 × 50 mm, 1.7 μm), and the column temperature was maintained at 40 °C during the analysis. The mobile phase consisted of MeCN (solvent A) and 0.1% formic acid in water (solvent B) at a flow rate of 0.4 mL/min. The gradient elution conditions were as follows: 0–1 min, 2–2% A; 1–1.5 min, 2–10% A; 1.5–7 min, 10–43% A; 7–8 min, 43–95% A; 8–9 min, 95% A; 9–10 min, 95–2% A. The injection volume was 2 μL. The detection wavelength was at 330 nm.

The PhGs were detected and quantified by multiple reaction monitoring (MRM) in negative ionization mode (ESI^−^). The MS parameters of the ionization source were as follows: source temperature, 150 °C; capillary voltage, 3.15 kV; desolvation gas temperature, 400 °C; desolvation gas flow rate, 800 L/h; and cone gas flow rate, 150 L/h. Other optimized parameters, collision energies, and cone voltages are shown in Table 1. All raw data were processed using MassLynx V4.1 workstation (Waters Corp., Milford, MA, USA).

### 3.7. Method Validation

The selectivity of the method was assessed by chromatograms of blank plasma, blank plasma spiked with working solution of the four PhGs at the lowest limit of quantification (LLOQ) together with working solution of IS, and test plasma acquired at 30 min after intragastric administration of AIH extract. The samples were prepared and pretreated in the same approaches as in Section 3.4 and Section 3.5. 

Various concentrations of calibration standards (2.0, 5.0, 10, 25, 50, 100, 200, 400, 800, and 1000 ng/mL acteoside; 0.2, 0.5, 1.0, 2.5, 5.0, 10, 20, 40, 80, and 100 ng/mL isoacteoside; and 0.4, 1.0, 2.0, 5.0, 10, 20, 40, 80, 160, and 200 ng/mL martynoside and crenatoside) were processed according to the above procedures for sample preparation. The calibration curve was constructed by plotting analyte-to-IS peak area ratio (y) versus the concentration (x, ng/mL) of analyte and fitted to linear regression (y = ax + b) using 1/x as the weighting factor. The calibration curves were acceptable only if their correlation coefficients (*r*^2^) represented linearity of 0.99 or greater. The calibration curve was established daily throughout the method development and pharmacokinetic analysis. The LLOQ was determined by testing the lowest analytical concentration of the calibration curve. 

The accuracy and precision of the method were evaluated by analyzing QC samples at three concentrations prepared as in Section 3.4 with six replicates. To determine the intra- and inter-day accuracy and precision, six replicates at each concentration level were analyzed for five consecutive days. Accuracy was expressed as relative error (RE, %) values within ±15%, and precision was described as relative standard deviation (RSD, %) values less than 15%. 

The extraction recoveries at different QC levels were investigated by comparing the mean peak areas of the PhGs pipetted into blank plasma before and after protein precipitation, respectively. The absolute matrix effect expressed as matrix factor (MF) was evaluated by comparing the peak areas of the PhGs pipetted into rat plasma after protein precipitation with those dissolved in the initial mobile phase solution. The relative matrix effect was assessed based on the peak areas of the PhGs pipetted into six different individual sources of rat plasma. The IS normalized MF was determined by the absolute MF of analyte over that of the IS.

The stabilities of the four PhGs were determined by using QC samples in different conditions. Freeze-thaw stability was assessed after three freeze-thaw cycles (from −80 °C to room temperature). Short-term stability was determined after exposure of the QC samples at room temperature (25 °C) for 10 h. Long-term stability was assessed by exposing the samples at −20 °C for 20 days. The samples stored in the autosampler at 4 °C for 24 h were used to evaluate the post-preparative stability. Each QC concentration level was prepared in six replicate samples. 

### 3.8. Pharmacokinetic Study

AIH extract was given to the six rats at a dose of 10.0 g crude herb/kg body weight by intragastric administration. Blood samples (200 μL) were collected from the fossa orbitalis vein before dosing and at the time points of 5, 15, 30, and 45 min and 1, 2, 4, 6, 8, and 12 h after administration and transformed into heparinized Eppendorf tubes. Then, the blood samples were centrifuged at 12,000 × *g*, 4 °C, for 10 min. Each rat plasma sample (50 μL) was prepared in the same approaches as in Section 3.5. 

### 3.9. Data Analysis

Drug and Statistics (DAS) 3.2.8 software (Shanghai University of Traditional Chinese Medicine, Shanghai, China) was applied to calculate the pharmacokinetic parameters (t_1/2_, t_max_, C_max_, AUC_0–t_, and AUC_0–__∞_) of the four PhGs. All data were shown as mean ± standard deviation (SD).

## 4. Conclusions

In this study, an accurate and sensitive UPLC-MS/MS method was established and validated for the simultaneously quantitative determination of four PhGs (acteoside, isoacteoside, martynoside, and crenatoside) in rat plasma. This method which was specific to PhGs had good linearity, high accuracy and precision, and no significant matrix effect. By the established method, the preliminary pharmacokinetic features were firstly elucidated for AIH represented by the four PhGs in rats after intragastric administration of AIH extract. It was concluded that these four PhGs manifested relatively distant double peaks on the concentration-time curves and different elimination half-lives although they shared similar molecular structures. The achieved pharmacokinetic parameters may provide primary data and a scientific basis for the further research on the pharmacokinetics of AIH.

## Figures and Tables

**Table 1:** The optimized mass spectrometry parameters of the PhGs and IS.

Components	Retention Time (min)	MRM Transitions (precursor→product)	Collision Energy (v)	Cone Voltage (v)
Acteoside	3.37	623.2→161.0	50	76
Isoacteoside	3.56	623.2→161.0	50	76
Martynoside	4.42	651.2→175.0	36	78
Crenatoside	3.54	621.2→161.0	44	72
Genistein (IS)	5.02	270.0→133.0	34	78

**Table 2:** Contents of the four PhGs in AIH (means ± SD).

Components	Contents (mg/g)
Acteoside	6.245 ± 0.723
Isoacteoside	0.822 ± 0.102
Martynoside	0.071 ± 0.023
Crenatoside	0.023 ± 0.008

**Table 3:** Regression equation and LLOQ for the four PhGs.

Components	Linear Regression Equation	*r* ^2^	Range (ng/mL)	LLOQ (ng/mL)
Acteoside	y = 0.000655 x − 0.001804	0.9979	2.0–1000	2.0
Isoacteoside	y = 0.000917 x − 0.000488	0.9935	0.2–100	0.2
Martynoside	y = 0.005864 x − 0.001393	0.9990	0.4–200	0.4
Crenatoside	y = 0.000592 x − 0.000034	0.9949	0.4–200	0.4

**Table 4:** Accuracy and precision of the four PhGs in rat plasma (*n* = 6).

Components	Concentration (ng/mL)	Accuracy (RE%)		Precision (RSD%)	
		Intra-day	Inter-day	Intra-day	Inter-day
Acteoside	2.0	4.36	5.53	7.24	6.17
	5.0	−5.26	−3.81	2.17	3.60
	50.0	−7.25	−4.89	7.32	2.37
	800.0	4.70	4.27	7.04	5.90
Isoacteoside	0.2	9.88	−4.86	6.42	3.80
	0.5	2.98	−0.98	5.53	8.46
	5.0	4.24	−4.38	8.35	11.90
	80.0	9.26	7.27	8.27	9.47
Martynoside	0.4	−7.24	4.18	5.54	10.00
	1.0	2.04	3.41	9.09	10.49
	10.0	−1.02	1.54	2.70	1.80
	160.0	1.44	−2.71	1.47	3.39
Crenatoside	0.4	9.43	6.63	9.72	6.42
	1.0	−8.53	−5.12	12.37	13.08
	10.0	−5.32	−8.92	2.08	8.10
	160.0	−2.95	−5.22	6.39	4.35

**Table 5:** The extraction recovery and matrix effects of the four PhGs in rat plasma (*n* = 6).

Components	Concentration (ng/mL)	Extraction Recovery		Absolute Matrix Effect		Relative Matrix Effect	IS Normalized MF	
		Mean (%)	RSD (%)	Mean (%)	RSD (%)	RSD (%)	Mean ± SD	RSD (%)
Acteoside	5.0	75.51	8.03	99.00	12.97	9.41	0.95 ± 0.06	7.62
	50.0	88.51	9.75	85.03	4.40	3.22	0.93 ± 0.03	3.11
	800.0	97.14	3.43	86.70	6.21	8.97	0.92 ± 0.02	6.23
Isoacteoside	0.5	98.50	11.47	95.66	9.83	10.11	1.00 ± 0.10	7.96
	5.0	70.56	1.48	89.52	6.38	3.98	1.02 ± 0.09	12.14
	80.0	71.01	2.76	88.11	5.34	2.32	1.04 ± 0.11	2.35
Martynoside	1.0	104.54	12.41	89.27	12.66	4.78	0.96 ± 0.05	9.76
	10.0	92.81	9.64	101.02	7.78	6.33	0.98 ± 0.09	4.12
	160.0	98.19	4.14	96.23	3.42	5.22	0.99 ± 0.08	10.35
Crenatoside	1.0	82.96	11.42	85.31	2.34	10.90	1.00 ± 0.08	8.62
	10.0	90.11	9.57	89.72	10.64	3.51	0.97 ± 0.04	10.69
	160.0	80.38	5.33	106.66	4.47	2.96	0.90 ± 0.03	7.62

**Table 6:** The stability of the four PhGs in rat plasma (*n* = 6).

Components	Concentration (ng/mL)	Freeze and Thaw		Short-Term		Long-Term		Post-Preparative	
		RSD (%)	RE (%)	RSD (%)	RE (%)	RSD (%)	RE (%)	RSD (%)	RE (%)
Acteoside	5.0	2.92	−1.58	2.17	−5.26	2.51	−2.45	4.68	−2.72
	50.0	4.60	−5.42	7.32	−7.25	8.01	−8.44	11.78	1.40
	800.0	6.32	1.50	7.04	4.70	6.49	−0.01	2.27	−2.16
Isoacteoside	0.5	6.59	0.61	7.53	2.98	7.43	−1.42	9.61	−2.70
	5.0	9.90	−1.65	11.69	4.24	10.32	2.20	10.68	1.88
	80.0	1.99	0.61	6.28	2.98	6.06	−1.42	7.32	−2.70
Martynoside	1.0	9.46	3.03	9.09	2.04	8.74	1.27	7.47	2.27
	10.0	3.13	−0.19	2.70	−1.02	2.82	−0.79	1.82	−0.66
	160.0	1.13	−2.71	1.47	1.44	1.69	−0.91	2.47	−0.60
Crenatoside	1.0	6.42	2.46	12.37	−8.53	6.36	0.71	9.38	−3.03
	10.0	10.87	−8.98	10.42	−5.32	9.94	−11.6	12.48	−10.95
	160.0	5.27	−3.37	6.39	−2.95	3.80	−4.97	6.02	−1.40

**Table 7:** Pharmacokinetic parameters for the four PhGs in rat plasma after intragastric administration (means ± SD, *n* = 6).

Parameter	Acteoside	Isoacteoside	Martynoside	Crenatoside
AUC_0-t_/(µg/L × h)	1826.3 ± 680.2	70.9 ± 26.9	23.6 ± 6.9	64.7 ± 14.5
AUC_0-∞_/(µg/L × h)	2243.1 ± 894.6	87.0 ± 40.0	39.5 ± 15.5	76.0 ± 30.0
t_1/2_/(h)	5.6 ± 3.4	4.6 ± 3.1	9.0 ± 2.7	3.4 ± 3.1
t_max_/(h)	0.3 ± 0.1	0.4 ± 0.2	3.1 ± 3.6	6.8 ± 1.1
C_max_/(µg/L)	356.9 ± 64.2	58.2 ± 15.0	4.0 ± 0.9	10.9 ± 0.9
